# Coverage of mammography imaging in and outside an organized breast cancer screening program – variation by age and sociodemographic groups

**DOI:** 10.2340/1651-226X.2024.40830

**Published:** 2024-10-29

**Authors:** Joanna Fuhrmann, Sirpa Heinävaara, Tytti Sarkeala, Milla Lehtinen, Maiju Pankakoski

**Affiliations:** a Faculty of Medicine, University of Helsinki, Helsinki, Finland; b Finnish Cancer Registry, Helsinki, Finland

**Keywords:** breast cancer screening, opportunistic screening, screening program

## Abstract

**Introduction:**

In recent decades, attendance to organized breast cancer screening has been decreasing in European countries. This could be partly due to an increase in the use of opportunistic screening. The aim of this study was to assess the coverage of imaging in and outside the screening program in Finland during the period of 1999–2018. We also compared the usage of imaging services across sociodemographic groups in the more recent years (2017–2018).

**Methods:**

Our initial data consisted of 1,159,000 screening-target-aged women (50–69 years) in 1999–2018 and 1,849,000 women aged 30–89 years in 2017–2018. Data on organized breast cancer screening program was drawn from the Finnish Cancer Registry and supplemented with comprehensive individual data on mammograms and ultrasounds performed outside the program.

**Results:**

Among the screening-aged women (50–69), a clear decline in the overall imaging coverage was observed during the study period (from 89 to 85%). The use of outside imaging increased slightly but not enough to compensate for the overall decrease. There were large differences in coverages between sociodemographic groups. Compared to manual workers and the unemployed, upper-level employees were around two times more active in using outside imaging (8.2% vs. 3.6% and 4.3%, respectively).

**Interpretation:**

Overall breast imaging coverage has slowly decreased during the 2000s, while outside imaging has increased slightly. The coverage of imaging in and outside the program largely followed the same trends, with the highest usage concentrating on higher socioeconomical groups, native speakers and highly educated women.

## Introduction

Attendance to organized breast cancer screening has been decreasing in most European countries, including Finland [1]. This could be partly due to an increase in the use of opportunistic screening, that is, imaging services outside the screening program [2]. In organized screening, women are systemically invited to participate, typically based on their age, in regular intervals. The population-based approach objectively assesses eligibility by considering population-level risk as well as other essential criteria, as standardized in the World Health Organization’s principles for screening [3, 4]. Opportunistic screening occurs on the initiative of the patient or their healthcare provider. This typically happens when the patient asks to be screened, has symptoms, or the healthcare provider, for some other reason, considers it should be done. This approach places greater responsibility on the individual. Participation may vary depending on subjective willingness, which can be affected by, for example, health literacy, cultural beliefs, and knowledge about the program [5].

Organized, population-based screening is offered by the public sector and is generally free of charge or only has a minimal fee for the attendee. In contrast, opportunistic screening is often performed in the private sector, often imposing costs on the patient. Because queues for non-urgent visits may be long in the public sector, imaging services outside the screening program may be more accessible to higher-income patients who can pay for a private-sector visit as soon as concern arises. It is therefore likely that attendance in organized and opportunistic screening differs by socioeconomic factors [6, 7]. This study reports for the first time the longitudinal coverage of imaging in and outside the screening program in Finland during the 2000s. For the most recent study years, we also assess inequities in imaging coverage by comparing the usage of these imaging services between sociodemographic factors.

## Materials and methods

In Finland, all women aged 50–79 are invited by personal letters to attend the organized, nation-wide breast cancer screening program every 2 years. Mammography is the primary screening method. If the results are abnormal, the patient is informed and called in for further testing and any possible following treatments through the program. Attendance is voluntary and cost-free.

Individual data on imaging outside the organized screening program was gathered for this study. The Radiation and Nuclear Safety Authority in Finland provided the list of permission holders for a mammography machine in 2017 with an update in 2019 [8]. We collected data on mammograms and ultrasounds outside the organized screening program from six private health service provider organizations and 19 hospital districts from 1999 onwards until 2018. Data request was denied at six private organizations and one hospital district. Approximately 30% of the imaging examinations were ultrasounds and 70% were mammograms. These data included imaging done for any reason, i.e. for screening or diagnostic purposes, and were independent of the examinations within the screening program. The data outside the screening program were merged at an individual level with the data on organized breast cancer screening program, drawn from the Finnish Cancer Registry.

**Figure 1:**
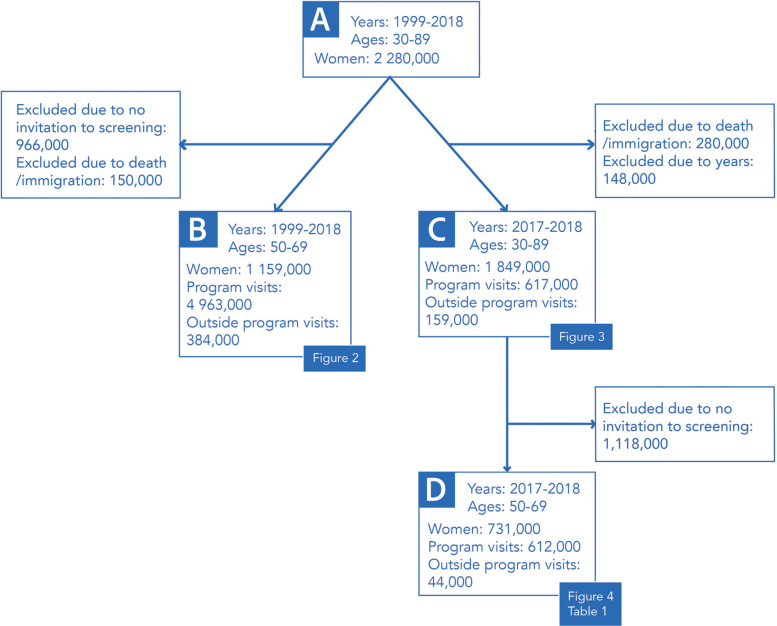
Flowchart depicting the data sets used in each analysis. Numbers are rounded to the nearest thousand.

We reported the temporal change in imaging coverage during 1999–2018 for the screening-aged women (50–69 years) (*n* = 1,159,000; B in Figure 1). To get a more detailed overview of the current situation, age and sociodemographic factors were examined for the most recent years 2017–2018. We looked at imaging coverages by ages 30–89 (*n* = 1,849,000; C in Figure 1). Coverages by sociodemographic variables were examined for women invited to the program (ages 50–69, *n* = 731,000; D in Figure 1). The time trend and sociodemographic factors were examined only among women invited to the screening program to get a clear picture of the distribution of imaging conducted within and outside the screening program. Data on socioeconomic status and education was provided by Statistics Finland, and data on language and home municipality was derived from the Digital Population and Data Services Agency.

Socioeconomic status was reported by employment status. The categories were employer or self-employed; higher employee; lower employee; manual workers; unemployed; retiree & student. Higher employees occupied higher organizational levels, whereas lower employees worked at the operational level with more practical tasks [9]. Retirees included both the elderly and people with early retirement due to illness, for example [10].

Education was reported by the last obtained level: primary education or unknown; secondary education; higher education. The classification was based on ISCED 2011 [11]. We only had information on secondary or higher education, and the rest were assumed to have completed the Finnish compulsory primary education.

We used two types of categorizing for language. The first grouped the participants into native or non- native speakers – native speakers being those whose mother tongue was a language native to Finland (Finnish, Swedish & Sami). The second type looked closer into the largest non-native language groups in Finland: Russian, English, Arabic, Chinese, Estonian, Thai, and Somali. The participants’ home municipalities were grouped into three types: urban, semi-urban, and rural. The classification was defined by Statistics Finland [12].

Our data on breast imaging coverage was categorized into four forms of imaging: no imaging, imaging only within the screening program, imaging only outside the program, and imaging both within and outside the program. Two-year coverages (with 95% confidence intervals) were calculated for each category, considering all imaging visits conducted during the index year and the preceding year. The time trend of the coverages among women invited to screening was reported for the years between 1999 and 2018. Coverages by age and sociodemographic variables were reported from 2017 through 2018. Numbers were presented for ages 30–89 by year in Figure 3. In addition, coverages by 5-year age group were reported in the text. The coverage comparisons between the sociodemographic variables (socioeconomic status, education, language, municipality type) were adjusted with age, using direct standardization and exact confidence intervals [13]. Statistical analyses were conducted using the R program (version 4.3.2). This study was approved by the Finnish Institute for Health and Welfare (permit no. THL/1795/14.06.00/2023). Data from Statistics Finland was used under a separate permit (TK-53-675-17). Written informed consent is not required for register-based studies in Finland.

## Results

### Temporal change

**Figure 2:**
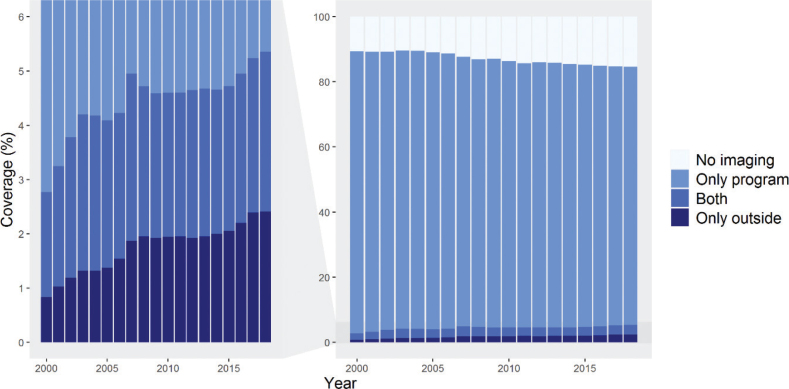
Two-year imaging coverages in women aged 50–69, residing permanently in Finland and invited to organized breast cancer screening from 1999 to 2018 (*N* = l,159,000). Each bar represents imaging visits conducted during the index year and the preceding year. The left-side graph is zoomed from the graph on the right.

Our initial data consisted of 2.3 million women aged 30–89 years and residing in Finland in 1999–2018 (A in Figure 1). After excluding those women who had died or emigrated within the 2-year coverage periods, we ended up with approximately 1,159,000 women aged 50–69 years and invited to take part in organized breast cancer screening in 1999–2018 (B in Figure 1). These women had altogether 4,963,000 imaging examinations within the program and 384,000 examinations outside of it. Figure 2 shows the 2-year imaging coverages in this group. A clear decline in overall imaging was observed, while the use of outside program imaging increased slightly during the study period. In 1999, the 2-year imaging coverage was 89.4%. During the depicted period, a decline of 4.8 percentage points was observed, leading to a coverage of 84.6% in 2018 (Figure 2).

The increase in the use of outside program imaging was not large enough to compensate for the overall decrease in the total coverage. The proportion of those undergoing both outside and within program imaging increased by 1 percentage point, from 1.9% in 1999 to 2.9%. The increase in the proportion of women who only used outside program imaging was steeper, increasing from 0.8% in 1999 to 2.4% in 2018 (Figure 2).

### Age

**Figure 3:**
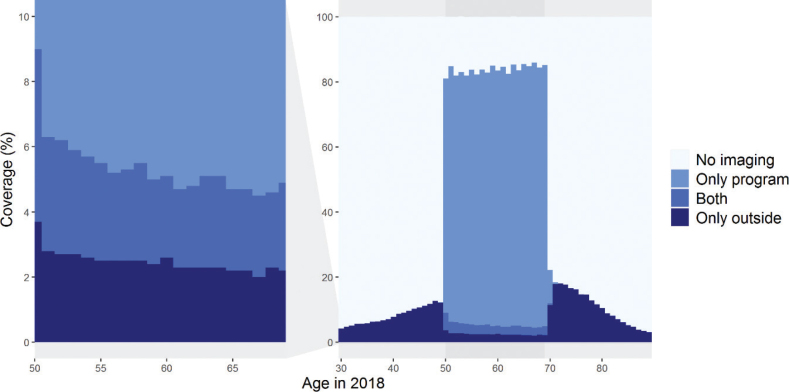
Two-year imaging coverages by age in women aged 30–89 in 2017–2018 (*N* = l,849,000). The left-side graph is zoomed from the graph on the right.

Figure 3 shows breast image coverages for ages 30–89 for the latest period in 2017–2018 (*n* = 1,849,000; C in Figure 1). These coverages were based on 617,000 imaging examinations within the program and 159,000 examinations outside of it. Within the screening age range, the overall imaging coverage increased with age: the highest coverage was in women aged 65–69 (85.2%), and the lowest in women aged 50–54 (82.6%). Conversely, younger screening-aged women were more likely to undergo outside program imaging than older women (6.6% at ages 50–54; 4.7% at ages 65–69) (Figure 3).

Women above the screening age were more likely to undergo breast imaging than women below the screening age. Coverage was the highest at the ages nearest to the screening target age: 45–49-year-olds and 70–75-year-olds underwent imaging more often (11.8 and 16.3%) than the ages below or above, respectively (Figure 3).

### Sociodemographic factors

The results on socioeconomic status, education, native language, and municipality type included 731,190 women aged 50–69 and invited to screening during 2017–2018 (D in Figure 1). They had altogether 612,000 imaging examinations within the program and 44,000 examinations outside of it. The imaging coverage in this data was in total 84.7% for any imaging, 79.3% for only program, 2.4% for only outside imaging and 2.9% for both, program and outside imaging (Table 1).

**Table 1:** Coverage in women aged 50–69 during 2017–2018 by sociodemographic group.

	Invited	Any imaging			Only program			Only outside			Both		
	*N*	Crude %	Adj. %	95% CI	Crude %	Adj. %	95% CI	Crude %	Adj. %	95% CI	Crude %	Adj. %	95% CI
Total	731,190	84.7			79.3			2.4			2.9		
Socioeconomic status*													
Employers and self-employed	34,973	85.4	85.4	84.3–86.5	78.9	79.2	78.1–80.3	3.1	3.1	2.9–3.3	3.4	3.1	2.9–3.3
Upper-level employees	92,910	88.5	88.6	87.9–89.3	80.3	80.6	79.9–81.3	3.8	3.7	3.6–3.8	4.4	4.2	4.1–4.4
Lower-level employees	200,321	89.1	89.3	88.8–89.8	83.5	83.9	83.4–84.4	2.3	2.3	2.2–2.4	3.3	3.1	3.0–3.2
Manual workers	70,762	83.6	84.0	83.3–84.8	80.0	80.5	79.8–81.2	1.6	1.6	1.5–1.7	2.0	2.0	1.8–2.1
Unemployed	41,556	75.5	76.5	75.7–77.4	71.2	72.3	71.4–73.2	2.1	2.1	1.9–2.2	2.2	2.2	2.0–2.3
Students	3,928	76.0	77.2	73.5–81.1	70.5	71.7	68.1–75.4	2.7	2.5	1.9–3.3	2.8	3.0	2.3–4.0
Pensioners	271,063	83.1	76.9	76.4–77.4	78.4	72.2	71.7–72.7	2.2	2.3	2.2–2.4	2.5	2.4	2.3–2.5
Unknown/others	15,677	63.4	64.6	63.2–66.0	58.5	59.9	58.6–61.3	2.6	2.5	2.2–2.8	2.3	2.2	1.9–2.4
Education													
Higher education	303,706	88.0	88.0	87.7–88.4	81.0	81.2	80.9–81.5	3.1	3.1	3.0–3.2	3.8	3.7	3.7–3.8
Secondary education	308,560	85.0	85.0	84.7–85.4	80.7	80.7	80.4–81.0	1.9	1.9	1.9–2.0	2.4	2.4	2.4–2.5
Primary or unknown**	118,924	75.4	72.8	72.3–73.4	71.5	68.8	68.3–69.3	1.9	2.0	1.9–2.1	2.0	2.1	2.0–2.2
Language													
Native	699,165	85.5	85.5	85.3–85.8	80.1	80.1	79.9–80.3	2.4	2.4	2.4–2.5	3.0	3.0	2.9–3.0
Non-native	32,025	65.9	65.9	64.9–66.8	61.7	61.9	60.9–62.8	2.1	2.0	1.8–2.1	2.2	2.0	1.9–2.2
Russian	12,884	74.2	74.1	72.6–75.7	70.2	70.3	68.8–71.8	1.8	1.7	1.5–2.0	2.2	2.1	1.9–2.4
Estonian	6,691	58.8	58.4	56.4–60.5	55.5	55.4	53.4–57.4	2.0	1.8	1.5–2.2	1.3	1.2	0.9–1.5
Thai	1,345	64.6	62.2	55.8–69.4	62.4	60.3	54.0–67.5	0.9	0.9	0.4–2.6	1.3	1.0	0.5–2.7
English	930	55.2	55.2	50.1–60.9	49.8	50.0	45.1–55.4	3.7	3.5	2.3–5.1	1.7	1.8	1.0–3.2
Chinese	850	62.4	60.1	54.1–66.8	58.9	57.2	51.3–63.8	1.5	1.2	0.5–2.9	1.9	1.7	0.9–3.3
Arabic	760	58.3	57.1	50.8–64.2	51.2	50.6	44.7–57.3	3.3	3.4	2.0–5.9	3.8	3.0	1.8–5.2
Somali	710	24.1	22.4	18.6–27.0	21.3	19.9	16.4–24.3	2.3	1.8	1.0–3.6	0.6	0.7	0.1–2.4
Other	7,855	64.9	63.9	61.9–65.9	59.1	58.6	56.7–60.5	2.8	2.5	2.1–2.9	3.0	2.9	2.5–3.3
Municipality type													
Urban	502,795	83.9	83.9	83.6–84.2	77.9	78.0	77.7–78.2	2.7	2.7	2.7–2.8	3.2	3.2	3.2–3.3
Semi-urban	107,515	86.7	86.7	86.2–87.2	82.3	82.3	81.8–82.8	1.9	1.9	1.8–2.0	2.5	2.5	2.4–2.6
Rural	120,880	86.2	86.2	85.6–86.7	82.6	82.5	82.0–83.1	1.5	1.5	1.5–1.6	2.1	2.1	2.0–2.2

Crude and age-adjusted percentages (direct standardization).

* Ten-year age groups were used for age-standardization for socioeconomic status. Five-years age groups were used for other variables.

** Information was only available for secondary/higher education, and the rest were assumed to have completed the Finnish compulsory primary education.

The age-adjusted coverage ranged from 64.6% (95% CI = 63.2–66.0) in those with unknown socioeconomic status and 76.5% (95% CI = 75.7–77.4) in the unemployed to 89.3% (95% CI = 88.8–89.9) in lower-level employees (Table 1). Clear differences in the age-adjusted imaging coverages in and outside the screening program were found between sociodemographic groups.

Upper-level employees were the most active in attending imaging outside the program. This group was also the most likely group to undergo imaging both in and outside the program (4.2%; 95% CI = 4.1–4.4%) and only outside program (3.7%; 95% CI = 3.6–3.8%). The lowest coverage in outside imaging was seen in manual workers (only outside 1.6%; 95% CI = 1.5–1.7%) and the unemployed (only outside 2.1%; 95% CI = 1.9–2.2%) (Table 1).

The overall imaging coverage increased with higher education. The highest coverage was found in the higher-educated women (88.0%; 95% CI = 87.7–88.4%), and the lowest in women with primary or missing education (72.8%; 95% Cl = 72.3–73.4%). Having only outside imaging was also more common in higher-educated women (3.1%; 95% CI = 3.0–3.2%) compared to women with primary or missing education (2.0%; 95% CI = 1.9–2.1%) (Table 1).

**Figure 4:**
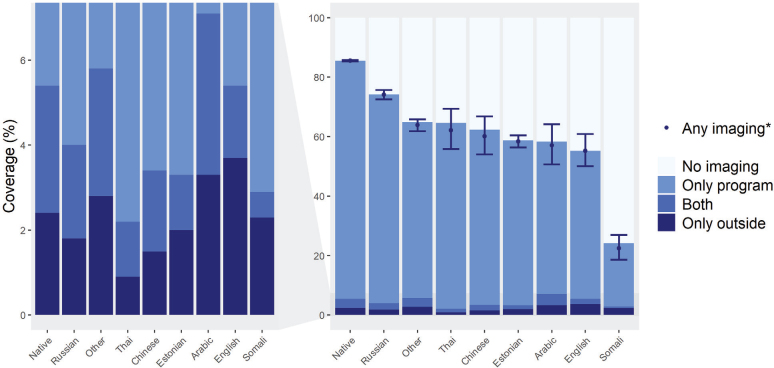
Two-year imaging coverages by mother tongue in 2017–2018 (*N* = 731,000). *Age-adjusted (direct adjustment). The graph depicts crude percentages, except for any imaging. Age adjusted percentages and 95% confidence intervals by form of imaging are reported in Table 1.

Substantial differences in participation were found when comparing women whose mother tongue was Finnish, Swedish or Sami to those whose was not. These native speakers had a much higher overall imaging coverage (85.5%; 95% CI = 85.3–85.8%) than non-native speakers (65.9%; 95% CI = 64.9–66.8%). The same pattern was seen in those only participating in program imaging with corresponding rates being 80.1% (95% CI = 79.9–80.3%) and 61.9% (95% CI = 60.9–62.8%), respectively (Table 1). When comparing different language groups within the non-natives, substantial differences were found, both in overall and in outside imaging (Figure 4). For example, Russian women attended program imaging actively and had the highest overall coverage of all non-native groups (74.1%; 95% CI = 72.6–75.7%). On the other side, Somali women had a poor overall coverage of 22.4% (95% CI = 18.6–27.0%) (Table 1 and Figure 4). In urban municipalities, the overall imaging coverage was lower (83.9%; 95% CI = 83.6–84.2%) than in semi-urban municipalities (86.7%; 95% CI = 86.2–87.2%) and in rural municipalities (86.2%; 95% CI = 85.6–86.7%). Conversely, outside program imaging was more common in urban municipalities (only outside imaging 2.7%; 95% CI = 2.7–2.8%) compared to semi-urban municipalities (only outside imaging 1.9%, 95% CI = 1.8–2.0%) or rural municipalities (only outside imaging 1.5%, 95% CI = 1.5–1.6%) (Table 1).

As a sensitivity analysis, we also examined the group with unknown/other socioeconomic status with respect to the other sociodemographic characteristics. A relatively large proportion in this group were foreign-language speakers compared to other groups (19% vs. 4%) and had primary or missing education (28% vs. 16%).

## Discussion

### Summary & interpretation of results

This study provided valuable insights into temporal, sociodemographic, regional, and age patterns of breast imaging in and outside the Finnish organized screening program. The data sheds light on the declining overall participation, which decreased from 1999 to 2018, going from 89 to 85%. In contrast to the overall decline in screening participation, imaging services outside the program gained popularity. This may have influenced the decline in overall participation. Nonetheless, the use of outside-program imaging was still low and only a small percentage of women underwent both in and outside program imaging.

There were distinct differences in screening participation depending on socioeconomic status, education, and native language. Groups within the workforce were more likely to attend the screening program than those outside of it. The same largely applied to outside program imaging. This could possibly be due to the latter being primarily offered in the private sector, resulting in expenses and thus being more accessible to individuals with a higher income or/and occupational health care. Earlier studies have found that high socioeconomic status is associated with the use of private or occupational health in Finland [14]. In addition, women with primary education or no education had lower coverages in all forms of imaging compared to higher-educated women. Similar patterns have been seen in earlier studies [15, 16].

The study revealed significant disparities between native and non-native speakers in imaging uptake. Non-native speakers, predominantly immigrants, exhibited lower uptake than the native speakers. This aligns with previous findings on reduced healthcare usage among immigrants due to language barriers or other internal factors [17, 18]. Previous research on screening participation in Nordic countries, including Finland, reported the same findings [19]. There were also language-specific differences in the non-native group.

Regionally, the study found lower participation in the screening program in urban municipalities compared to rural and semi-urban areas, similar to the patterns of earlier studies in Denmark [20]. However, the usage of outside program imaging was higher in urban areas, likely influenced by the higher prevalence of private actors in larger cities and income polarization.

### Implications

It is noteworthy that breast cancer screening participation rates vary significantly between countries. Several countries have reported participation rates below 50%, while only three countries have achieved rates above 75% as of 2018 [21, 22]. While the overall participation in the screening program in Finland is well over the acceptable EU level of 70% for program effectiveness [23], the trend is clearly negative. Action should be taken to reverse the trend, by targeting specific groups with low attendance such as those with lower education and groups outside the workforce.

According to our results, outside program imaging increased in Finland during the 2000s, but it was unlikely the reason for the decline in the attendance rate of the screening program. Outside imaging was, however, quite uncommon due to the well-established screening program. The situation may be different, e.g. in countries with more recently implemented screening programs [24].

The Finnish law states that a doctor’s (or dentist’s) referral is required for all medical procedures that expose the patient to radiation. Referrals are only to be made when it is medically warranted – the doctor is legally required to acquire all the information he or she needs to make the clinical decision that the potential good of the radiation outweighs the potential bad. This includes mammographies, but also ultrasounds and other diagnostic imaging used in breast cancer screening [25]. Consequently, a patient cannot attend outside program imaging without first consulting a doctor and therefore, none of these visits can be seen as directly unnecessary. Even so, it could possibly be beneficial to redirect some of the groups with higher outside program imaging uptake to attend the screening program. There is still concern about possible overlap and therefore futile exposure to imaging and unnecessary costs.

The reasons behind women choosing to use services outside the program are not known in the Finnish setting. Invitations to the organized screening program are sent to the whole target population, regardless of earlier screening activity, and often with a pre-booked appointment. Higher socioeconomic groups probably use outside program imaging for practical reasons (more available times, faster results, nicer facilities), as the price might not be as relevant. On the other hand, some groups with lower socioeconomic status, for example, a large part of non-native speakers, might do so due to a lack of knowledge about the organized screening program.

Sadly, but not surprising, we found low imaging coverage in the non-native speakers. Especially, Somali, Arabic and Estonian speakers should be targeted. Screening providers should work to improve this by acknowledging the potential barriers foreigners can experience for healthcare, such as lacking awareness of the health care system and limited language skills. Both language and culturally adapted interventions should be taken into account [26–28]. The European Commission has published guidelines for how to target socially disadvantaged women, which could be implemented to a higher extent [29].

### Strengths and limitations

In this study, we were able to assess the overall coverage of breast imaging, also outside the screening program, in Finland. Our study fills a critical gap as it provides an unprecedented overview of trends in outside program imaging coverage. The utilization of large and diverse data in this study enhanced the statistical power and precision of our analyses. The extensive, population-based data allowed for a detailed examination of the socio-economic patterns, leading to robust and reliable conclusions. The observed socioeconomic and regional trends in our study aligned with previous findings reported in European studies, validating the consistency of our results.

One of the limitations of this study is that breast cancer screening underwent gradual expansion over the study period. From 2007 onwards, the program, that had previously screened women aged 50–59, expanded with 10 years to include all women aged 50–69. Municipalities could implement this expansion gradually as they wished during the 10 following years. In 2016, the expansion was finalized [30]. Variations in coverage rates may have been influenced by this, although we used the invited population as the denominator in the time trend analysis. We did an additional analysis adjusting for age and that did not change the estimate meaningfully. The data on sociodemographic factors was limited to 2 years in our study. Consequently, our analysis may not have captured the broader dynamics and changes that could have occurred over a more extended period.

One hospital district and six private health provider organizations declined our request for data. These were all small units, and it is unlikely that this lack of information has introduced significant bias to our results. In the cross-sectional survey with the response rate of 98% for 2018, Radiation and Nuclear Safety Authority reported approximately 311,000 and 85,000 images taken in and outside the breast cancer screening program, respectively [2]. This implies that there should have been approximately 170,000 images taken outside the screening program in our data, whereas 159,000 were observed in 2017–2018. Our data thus covered the vast majority of images taken outside the screening program, but the overall coverage estimates tended to be slightly underestimated. Despite this underestimation, the magnitude of imaging outside the screening program, especially in the target age, is low in Finland. The data outside the program were not distinguished between imaging done for screening purposes and diagnostic imaging, as our main purpose was to get an overall picture of the magnitude of the service use. Thus, the nature of the imaging in and outside the program was not directly comparable. In addition, it is possible that women have sought care from the private sector during the screening episode. However, to our understanding, this is quite an uncommon phenomenon in breast cancer screening.

## Conclusion

We found that the overall breast imaging coverage was slowly decreasing in the 2000s, while outside program imaging increased. The increase in outside program imaging was not large enough to compensate for the decrease in screening participation. The coverage of opportunistic and organized imaging largely followed the same trends, the highest coverage rates concentrating on higher socioeconomical groups, native speakers, and highly educated women. Regionally, we found lower overall attendance for urban areas compared to semi-urban and rural areas. Outside program imaging, however, was more common in urban areas. Understanding these factors can inform targeted interventions to improve screening participation and promote better healthcare equity.

## Data Availability

The data generated in this study are not publicly available due to availability compromising patient confidentiality. Data are available from the Finnish Cancer Registry for researchers who meet the criteria for access to confidential data (i.e. research permit via Findata).
